# The Role of Overexpressed Apolipoprotein AV in Insulin-Resistant Hepatocytes

**DOI:** 10.1155/2020/3268505

**Published:** 2020-04-21

**Authors:** Wang Zhao, Yaqiong Liu, Xiaobo Liao, Shuiping Zhao

**Affiliations:** ^1^Department of Cardiovascular Medicine, The Second Xiangya Hospital, Central South University, No. 139 Middle Renmin Road, Changsha, Hunan 410011, China; ^2^Department of Cardiovascular Surgery, The Second Xiangya Hospital, Central South University, No. 139 Middle Renmin Road, Changsha, Hunan 410011, China

## Abstract

In this paper, we sought to explore the relationship between apolipoprotein AV (*APOAV*) overexpression and insulin resistance in hepatocytes. The insulin-resistant HepG2 cell model was constructed, and then, *APOAV*-overexpressed HepG2 cells (B-M) were induced by infecting with a recombinant adenovirus vector. Microarray data were developed from B-M samples compared with negative controls (A-con), and the microarray data were analyzed by bioinformatic methods. *APOAV*-overexpression induced 313 upregulated genes and 563 downregulated ones in B-M sample. The differentially expressed genes (DEGs) were significantly classified in fat digestion and absorption pathway. Protein-protein interaction network was constructed, and *AGTR1* (angiotensin II receptor type 1) and *P2RY2* (purinergic receptor P2Y, G-protein coupled 2) were found to be the significant nodes closely related with G-protein related signaling. Additionally, overexpression of *APOAV* could change the expression of Glut4 and release the insulin resistance of hepatic cells. Thus, *APOAV* overexpression may prevent the insulin resistance in liver cells by mediating the genes such as *AGTR1* and *P2RY2*.

## 1. Introduction

Insulin resistance has become an increasingly common metabolic syndrome in people around the word. The main understanding of the mechanism for insulin resistance is the glycometabolism disorder in liver [1]. Insulin resistance results in excessive glucose consumption in liver, which contributes to the development of hyperglycemia [2]. In addition, hyperglycemia is frequently associated with metabolic syndromes such as hypertension, coronary heart disease, and diabetes [3, 4]. Type 2 diabetes (T2D) is a common metabolic disorder that characterizes high blood sugar, insulin deficiency, and insulin resistance. T2D (non-insulin-dependent) is the risk for cardiovascular disease that is the main cause for death of diabetes patients [5].

Recently, apolipoprotein AV (APOAV), also served as the APOA5, has been found to be a novel apolipoprotein and to play a key role in the processes of insulin resistance and lipid metabolism [6, 7]. APOAV is mainly expressed in liver and closely associated with high-density lipoprotein [8]. Moreover, APOAV is also found to participate in the metabolism of triglycerides by stimulating hydrolysis of triglycerides, reducing the lipoprotein production rate, and facilitating clearance of triglyceride-rich lipoprotein [9]. A previous study suggests that *APOAV* is downregulated in insulin-treated mice and the expression of *APOAV* a contributor for hypertriglyceridemia [10]. The decreased expression *APOAV* is involved in insulin resistance-related hypertriglyceridemia [6]. *APOAV* seems to be closely related with insulin resistance in diabetics, but whether the overexpression of *APOAV* could prevent insulin resistance needs to be further determined.

Microarray technology has been widely used to generate the gene expression data on a genomic scale [11]. In this paper, we performed the microarray analysis for the human hepatoma (HepG2) cells to discover the differentially expressed genes (DEGs) induced by *APOAV* overexpression. HepG2 cells, which are characterized by the common physiological function in glucose metabolism with normal hepatic cells [12], were used in this study. Therefore, in the present study, we sought to explore the potential mechanism of relationship between *APOAV* expression and insulin resistance in hepatocytes.

## 2. Materials and Methods

### 2.1. Insulin-Resistant HepG2 Cell Model Construction

HepG2 cell lines (Cell Culture Center of Peking Union Medical Science, Beijing, China) were cultured at 37°C in Dulbecco's modified eagle medium (DMEM) supplemented with 10% (v/v) FBS (fetal bovine serum) and 100 IU penicillin/streptomycin/amphotericin in a humidified 5% CO_2_ atmosphere. When entering the logarithmic phase, the cells were washed by PBS, and then digested with trypsin enzyme. After the cells turned round, cell suspension was put into 15 ml centrifuge tube and centrifuged at 1000 rpm for 3 min and inoculated in 96-well plates at the density of 5 × 10^3^ cells/cm^2^.

In order obtain the insulin-resistant HepG2 cell model, the cells were treated with insulin at a series of concentration gradients. The insulin-resistant HepG2 cell model was induced as described previously [13]. Briefly, HepG2 cells were subjected to 24h-culture of 5 × 10^−5^, 5 × 10^−6^, 5 × 10^−7^, and 5 × 10^−8^ mol/L bovine insulin, followed by incubating in 10^−9^ mol/L insulin for 24 h. And to determine whether the insulin model was successfully constructed, glucose uptake rate and liver glycogen synthesis were measured. For glucose uptake rate measurement, the supernatant was absorbed into a 15 mL centrifuge tube at 1500 rpm/6 min, and transferred to a 1.5 ml EP tube. After the supernatant was diluted 6 times with distilled water, and the glucose uptake rate was determined by the urine glucose assay kit-oxidase assay according to the manufacturer's instructions. Glucose uptake rate = (preinduced glucose concentration-postinduced glucose concentration)/preinduced glucose concentration. For glycogen synthesis measurement, (1) the cells were digested with trypsin, centrifuged (3000 g, 5 min) and weighed; (2) hydrolysis: sample weight (mg): lye volume (*μ*L) = 1 : 3, boiled in boiling water for 20 min; (3) liver glycogen detection solution (1%); (4) the standard glucose solution: the standard glucose solution was prepared into 25, 12.5, 6.25, 3.125, and 1.5625 g/ml with distilled water; (5) OD620 nm value of each tube was measured; and (6) standard curve was drawn for calculating the liver glycogen concentration.

### 2.2. Western Blot Analysis

To determine the native APOAV expression in the induced model cells and insulin-resistant HepG2 cells infected by recombinant adenovirus, western blot analysis was performed. Briefly, the cells were lysed in a radioimmunoprecipitation assay (RIPA) buffer supplemented with for proteins extraction, and total protein was quantified with the bicinchoninic acid (BCA) assay. Total proteins (30 *µ*g/lane) were separated via 12% SDS-PAGE and then transferred to a polyvinylidene diﬂuoride (PVDF) membrane. After blocked with 5% skimmed milk for 1 h, the membrane was incubated with primary antibodies (APOAV5, 1 : 1000; beta actin, 1 : 1000) at 4°C overnight. Following washing with TBST for 3 times, the membrane was incubated with secondary antibody (antirabbit IgG-HRP, 1 : 5000; antimouse IgG-HRP, 1 : 5000) for 2 h at room temperature. Finally, the proteins were visualized using the ECL chemiluminescence reagent.

### 2.3. Recombinant Plasmid Vector with APOAV

The *APOAV* gene was amplified by PCR with the primers, such as 5′-ACACGGATCCATGGCAAGCATGGCTGCCGT-3′ (forward) and 5′-ACACGAATTCTCAGGGGTCCCCCAGATG-3′ (reverse). After the plasmid pHBAd-MCMV-GFP was digested with BamH1 and EcoR1, the recombinant plasmid vector was constructed by connecting with *APOAV* at 4°C overnight. The positive recombinant clone was named as pHBAd-MCMV-APOAV-GFP. The expressions of *APOAV* and GFP were regulated by cytomegalovirus (CMV) promoter.

### 2.4. Insulin-Resistant HepG2 Cells Infection by Recombinant Adenovirus

HEK293 was the common cell for packing the recombinant adenoviruses because of its E1 genes. To generate recombinant adenovirus vectors with *APOAV* expression, the pHBAd-MCMV-APOAV-GFP and adenovirus skeleton plasmid vector of pHBAd-BHG were coinfected into HEK293 cells with the lipofectamine 2000 kit following the manufacturer's instruction. After 6 h transformation, the recombinant adenoviruses were harvested. Then, the insulin-resistant HepG2 cells were cultured and infected with the recombinant adenovirus vector for 48 h. The successful infection by recombinant adenovirus was determined, when the expression of GFP was observed by fluorescence microscopy.

### 2.5. RNA Isolation

Total RNA of insulin-resistant HepG2 cells with APOAV overexpression (B-M) and negative controls (A-con) was isolated using TRIzol reagent (Invitrogen, Carlsbad, CA) according to the manufacturer's protocol. The expected quality of RNA was determined by measuring the absorbance ratios (A260/A280) between 1.8 and 2.0 under the spectrophotometer.

### 2.6. Microarray Analysis

The RNA library was constructed by using the NEBNext ultra directional RNA library prep kit for illumina (New England Biolabs, Ipswich, MA, USA) following the manufacturer's instruction. After the library quality was assessed by Aglient 2100 Bioanalyzer (Agilent Technologies, Palo Alto, CA), the next-generation sequencing was performed based on the Illumina HiSeq4000 platform (Illumina Inc., San Diego, CA). Read counts were normalized by TMM (trimmed mean of *M* values) in the edgeR package [14]. Then, the read count data were transformed to log2-counts per million (logCPM) for gene expression by the limma-voom package [15]. The differential gene expression analysis between B-M and A-con cells was carried out by the limma package in *R* [16, 17]. Genes with *P* < 0.05 and log_2_|fold change| ≥ 1 were considered to be significantly different. Cluster analysis for DEGs was performed by gplots in *R* [18].

### 2.7. Function Analysis

The functionally associated genes were classified with the aim to explore the altered function and pathways in B-M cells. The GO (Gene Ontology) functions and KEGG (Kyoto Encyclopedia of Genes and Genomes) pathways were analyzed following the protocol of DAVID (version: 6.8) [19]. The count ≥2 and *P* value < 0.05 were set as the cutoff value.

### 2.8. Protein-Protein Interaction Network Analysis

STRING (Search Tool for the Retrieval of Interacting Genes/Proteins) database is a collection of protein interaction pairs, including physical and functional interactions [20]. The gene interactions analysis was performed based on protein interactions by STRING version: 10.0 [21]. The protein-protein interaction (PPI) network was constructed by protein interactions with PPI score ≥ 0.4 and visualized by Cytoscape (version: 3.2.1) [22]. Then, the node degrees were calculated for screening the hub nodes.

### 2.9. Module Analysis

Cytoscape plugin ClusterONE [23] provided network cluster analysis for screening significant modules. Modules with *P* < 1.0*E* − 4 were considered to be significant. The GO functions and KEGG pathways significantly enriched by module genes were further analyzed by DAVID (version: 6.8) software. Subsequently, the module genes with high node degrees were screened out as feature genes.

### 2.10. Disease-Associated Gene Analysis

The Comparative Toxicogenomics Database (CTD, http://ctdbase.org/) [24] provides the gene-disease relationships recorded in the previous studies. All of the marker genes associated with T2D were retrieved from the CTD database.

## 3. Verification Experiments

### 3.1. AML12 Cells with *APOAV* Overexpression

Mouse liver cells of AML12, purchased from the Chinese Academy of Sciences, Shanghai, China, were divided into the blank group, normal control group (empty vector), and *APOAV* group (*APOAV* overexpression). The recombinant plasmid vectors with *APOAV* overexpression were constructed according to the method mentioned above. Then, the recombinant plasmid vectors were transfected into AML12 cells. The AML12 cells successfully infected with the recombinant adenovirus vector were collected under fluorescence microscopy.

### 3.2. RT-qPCR

Total RNA from AML12 cell lines was extracted according to the method mentioned above, and the expression level of *APOAV* was determined according to the qRT-*PCR* with the following amplification conditions: predegeneration at 95°C for 3 min, followed by 40 cycles at 95°C for 30 sec, 30 sec at 60°C. *GAPDH* was used as the internal control, and the primer sequences were as follows: APOAV: forward, 5′-TCCTCGCAGTGTTCGCAAG-3′, and reverse, 5′- GAAGCTGCCTTTCAGGTTCTC-3′; GAPDH: forward, 5′ -GGTGAAGGTCGGTGTGAACG-3′, and reverse, 5′-CTCGCTCCTGGAAGATGGTG-3′. All reactions were performed in triplicate, and relative expressions of *APOAV* in cell lines were calculated by the 2^−ΔΔct^ method.

### 3.3. Enzyme-Linked Immunosorbent Assay (ELISA)

After infected with recombinant adenovirus vectors, the cells were adjusted to 5 × 10^4^ cells/cm^2^ and then maintained at 37°C overnight. When the cells grew up to 50%, the cells were treated with 5 × 10^−7^ mol/L insulin for 48 h. The level of glucose was evaluated by the ELISA kit (Beijing Pulilai gene co. LTD, Beijing, China) following the manufacturer's protocol.

Furthermore, the levels of Glut4 in the three groups were measured with the ELISA kit (Shanghai Enzyme-linked Biotechnology Co., Ltd, Shanghai, China) according to the manufacturer's protocol.

### 3.4. Statistical Analysis

All the data were expressed as mean ± SD (standard deviation). Statistical analyses were performed with Graphpad prism 5 (Graphpad Software, San Diego, CA). The comparison among groups was performed by one-way ANOVA (one-way analysis of variance). Differences between groups were compared by LSD (least significant difference test) method. *P* < 0.05 was considered significant.

## 4. Results

### 4.1. The Construction of Insulin-Resistant HepG2 Cell Model

**Figure 1:**
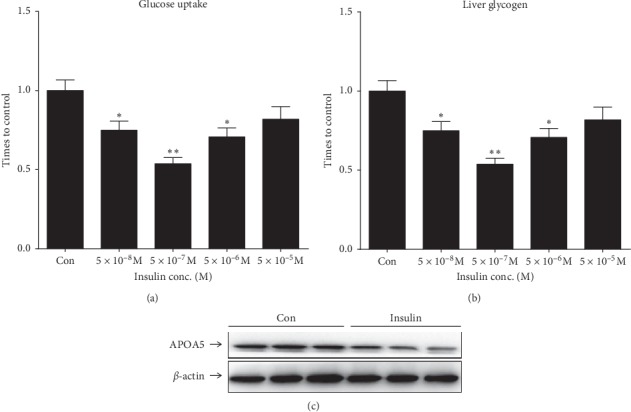
Insulin-resistant HepG2 cell model construction. (a) Glucose uptake rate and (b) liver glycogen synthesis after HepG2 cells treated with 5 × 10^−5^, 5 × 10^−6^, 5 × 10^−7^, and 5 × 10^–8^ mol/L bovine insulin. (c) APOAV protein expression in insulin-resistant HepG2 cells.

As illustrated in Figures 1(a) and 1(b), compared with normal control, the insulin groups with 5 × 10^−8^, 5 × 10^−7^, and 5 × 10^−8^ could significantly decrease the glucose uptake rate and liver glycogen synthesis of HepG2 cells. Specially, the insulin concentration of 5 × 10^−7^ was the most significant effect, which was selected for the following experiment. Furthermore, the glucose uptake rate and liver glycogen synthesis were used as the criteria for insulin-resistant cell model construction. Moreover, the results of western blot analysis showed that APOAV 5 levels in insulin-resistant HepG2 cells were decreased in comparison to normal controls (Figure 1(c)).

### 4.2. Insulin-Resistant HepG2 Cells Infected with Recombinant Adenoviral Vectors

**Figure 2:**
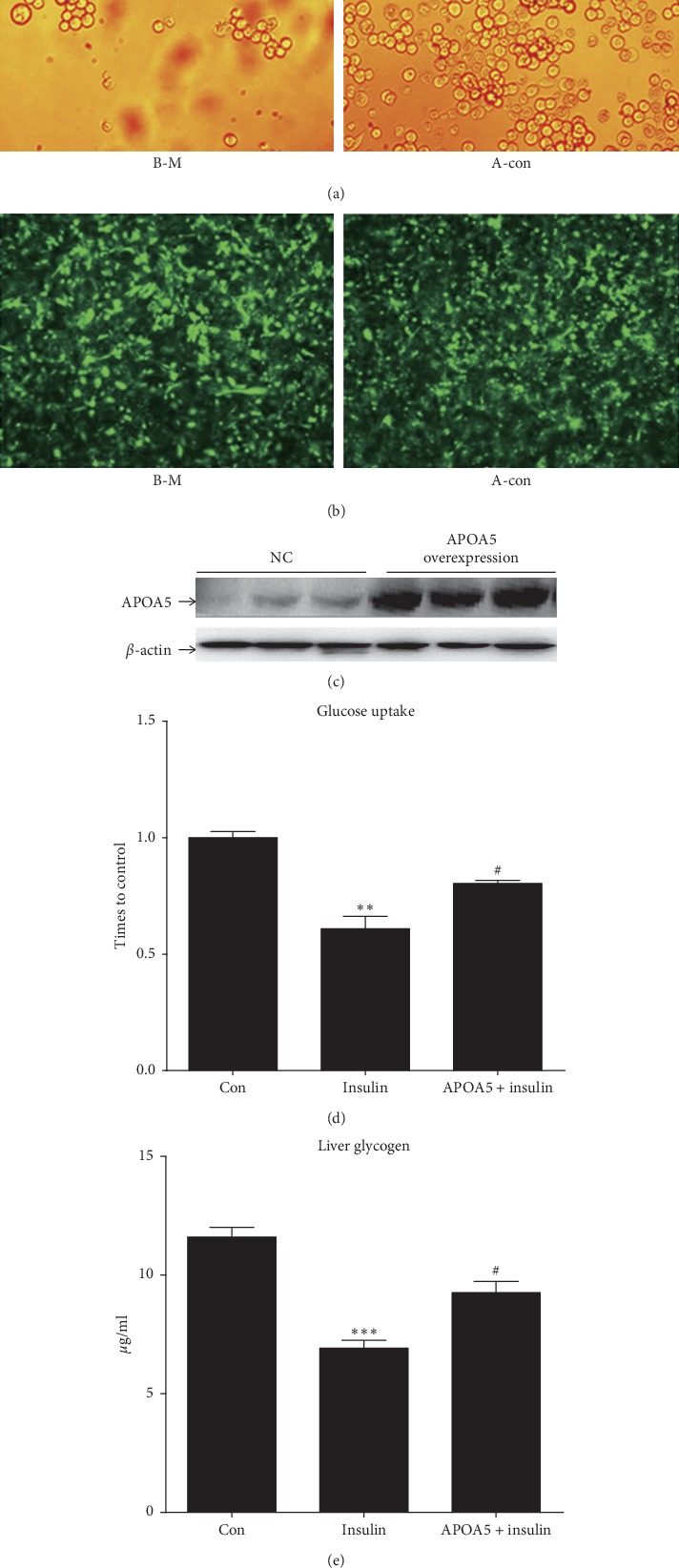
Successful construction of the insulin-resistant HepG2 cells with APOAV overexpression. (a) Recombinant adenoviruses packing HEK293 cells were infected with recombinant adenoviruses with APOAV overexpression. (b) The fluorescence images of insulin-resistant HepG2 cells with APOAV overexpression. B-M: insulin-resistant HepG2 cells with APOAV overexpression; A-con: negative controls. (c) Western blot analysis for APOAV 5 expression in control and insulin-resistant HepG2 cells with APOAV overexpression. (d) Glucose uptake rate and (e) liver glycogen synthesis in normal control, insulin-resistant HepG2 cells, and insulin-resistant HepG2 cells with APOAV overexpression.

HEK239 were independently infected with APOAV overexpressed recombinant adenoviruses and GFP control. As shown in Figure 2(a), the cells changed to round and plaques appeared obviously, which illustrated that the recombinant vectors were constructed successfully. After adenovirus vectors transfection, the GFP-positive cells were detected with the green fluorescence (Figure 2(b)). Moreover, compared with the controls, the expression of *APOAV* 5 protein in insulin-resistant HepG2 cells infected by recombinant adenovirus was upregulated (Figure 2(c)). In comparison to the insulin-resistant HepG2 cells, the glucose uptake rate and liver glycogen synthesis in cells infected by recombinant adenovirus were significantly increased (both, *P* < 0.05; Figures 2(d) and 2(e)), respectively. All these suggested that insulin-resistant HepG2 cells were successfully infected by recombinant adenovirus.

### 4.3. Identification of DEGs

**Figure 3:**
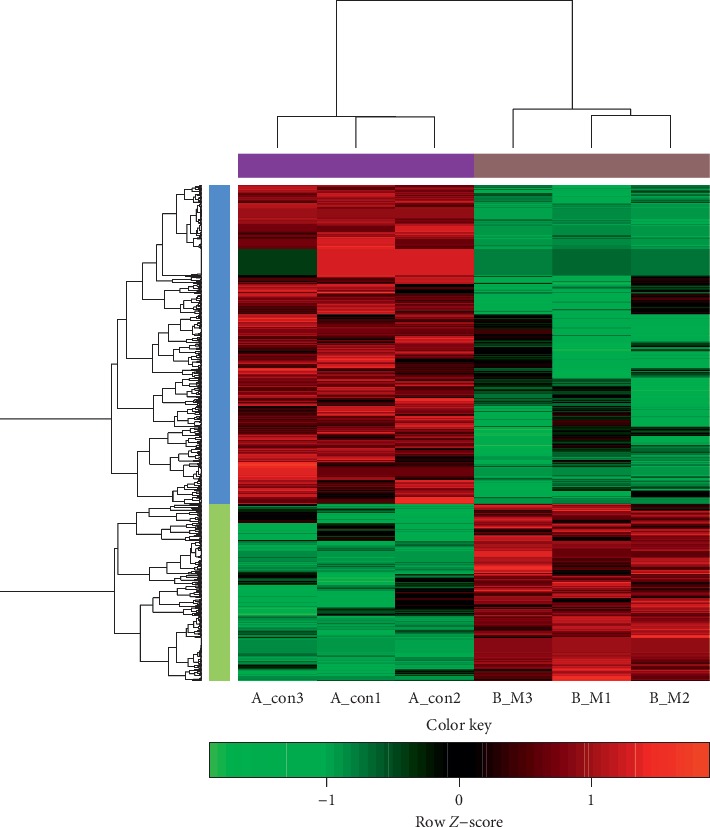
Hierarchical clustering analysis for differentially expressed genes. The color scale at the top illustrates the relative expression level of an mRNA. Red represents a high relative expression and blue represents a low relative expression.

The gene expression difference between B-M and A-con cells was assessed by microarray array analysis. The comparison of the gene expression pattern for B-M and A-con cells showed 876 genes differentially expressed (*P* < 0.05 and log_2_|fold change| ≥ 1), of which 313 were upregulated and 563 were downregulated. The hierarchical clustering were performed in two dimensions such as samples and genes (Figure 3), suggesting the clear differentiation was observed between B-M and A-con samples.

### 4.4. Characterization of Altered Function and Pathways

In order to analyze the potential biological function that was interfered by genes differentially expressed, the DEGs were annotated based on GO and KEGG databases. Results showed that the significantly different genes were classified in different GO categories (BP: biological process, CC: cellar component, and MF: molecular function) and pathways. The upregulated genes were closely related with intrinsic apoptotic signaling pathway by p53 class mediator-related BP, synaptic vesicle membrane-related CC, and calcium-dependent phospholipid binding-related MF. The downregulated genes were enriched in cell-cell signaling-related BP, extracellular region-related CC, and lipid transporter activity-related MF. In addition, the cancer-associated pathways were significantly associated with upregulated genes, such as small cell lung cancer and prostate cancer. The downregulated genes showed close association with systemic lupus erythematosus, ABC transporters, and legionellosis pathways (Table 1). And all these data reveal the potential processes and pathways that DEGs might be involved.

### 4.5. PPI Network and Hub Nodes Construction

**Figure 4:**
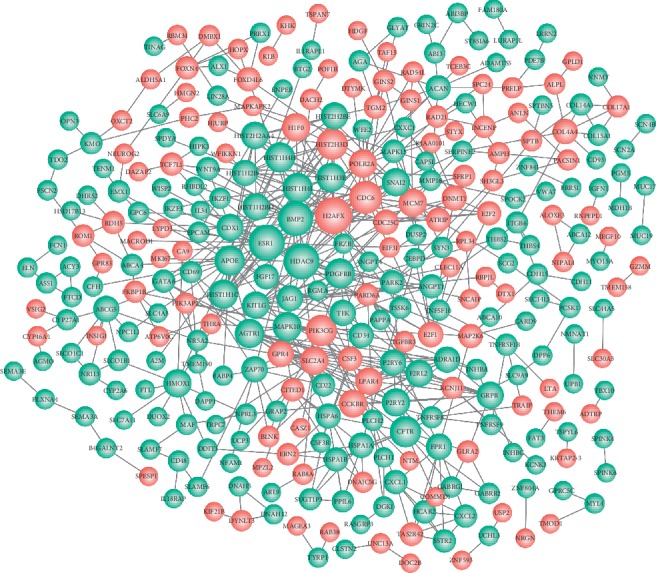
Protein-protein interaction (PPI) network construction for differentially expressed genes. A total of 317 nodes and 547 edges were identified. Red, upregulated genes; green, downregulated genes.

Total 547 protein interaction pairs with PPI score ≥0.4 were identified in the PPI network. As shown in Figure 4, the PPI network was constructed, which was comprised of 317 nodes and 547 edges. The hub nodes with degree ≥10 in network were screened out (Table 2), such as H2AFX (H2A histone family member *X*, degree = 19), HDAC9 (histone deacetylase 9, degree = 18), ESR1 (estrogen receptor 1, degree = 18), BMP2 (bone morphogenetic protein 2, degree = 17), and CDC6 (cell division cycle 6, degree = 15), which could be selected as the key genes.

### 4.6. Significant Modules in PPI Network

**Figure 5:**
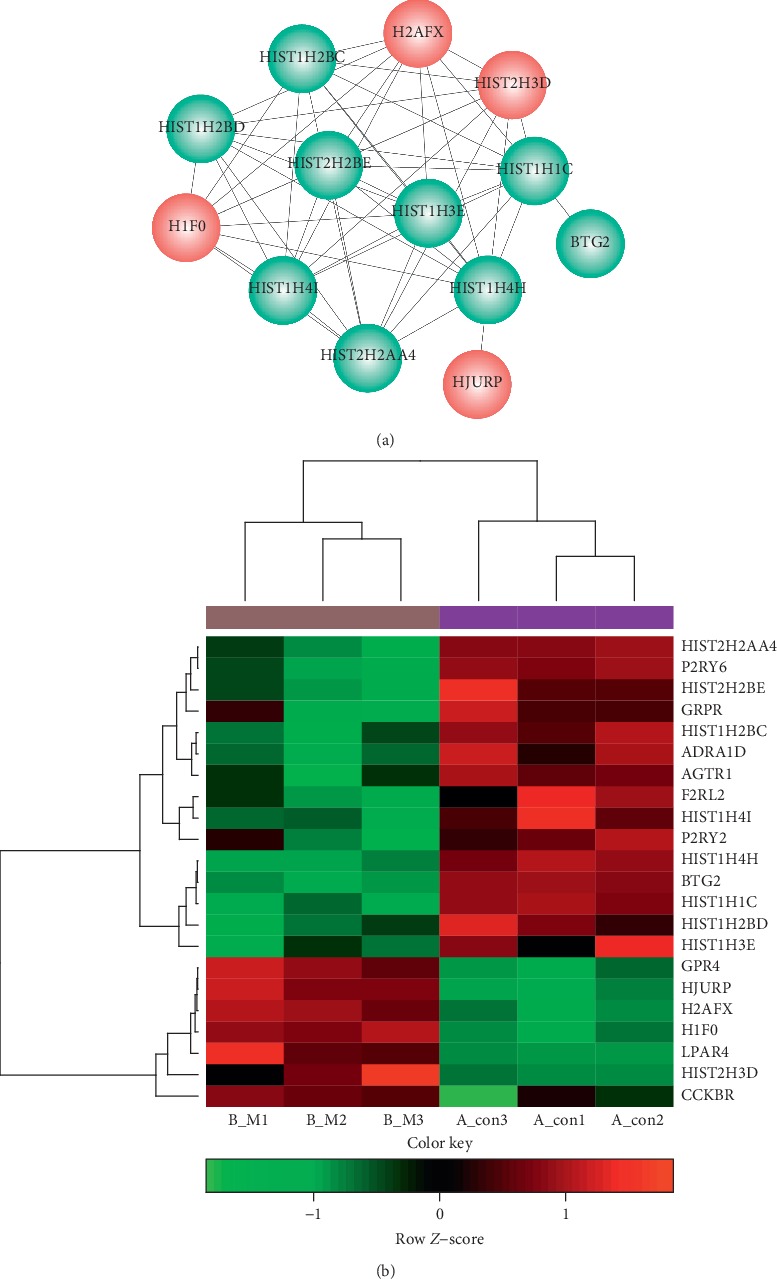
(a) Functional modules screened in PPI network. Red, upregulated genes; green, downregulated genes; PPI, protein-protein interaction. (b) Hierarchical clustering analysis for module genes. The samples of insulin-resistant HepG2 cells with APOAV overexpression (B-M) and negative controls (A-con) were distinguished clearly by the expression profiles of differentially expressed genes.

With the application of ClusterONE software, two modules were found to be significant. As shown in Figure 5(a), there were 9 nodes in module 1 and 13 nodes in module 2. Most of the module genes showed high node degrees in PPI network (Table 3). In addition, hierarchical clustering revealed the existence of 2 group samples (B-M and A-Con cells) were separated clearly by module genes (Figure 5(b)). All these results determined that the DEGs in modules were the feature genes for B-M and A-Con cells.

DAVID tool summarized the biological importance of the group of functionally related genes with an enrichment score. Function classification for genes in module 1 revealed a series of items, such as alteration of phospholipase C-activating G-protein coupled receptor signaling pathway (GO:0007200), integral component of plasma membrane (GO:0005887), G-protein-coupled purinergic nucleotide receptor activity (GO:0045028), and neuroactive ligand-receptor interaction pathway (hsa04080). Similarly, the module 2 genes were sorted into function classifications, including the nucleosome assembly (GO:0006334), nucleosome (GO:0000786), protein heterodimerization activity (GO:0046982), and systemic lupus erythematosus (hsa05322) pathway (Table 4).

### 4.7. Verification Experiments

#### 4.7.1. APOAV Expression Promoted the Glucose Absorption and Glut4 Levels of AML12 Cell

**Figure 6:**
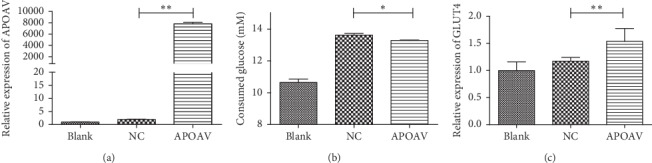
(a) APOAV expression in the blank group, NC group, and APOAV overexpression group measured by qRT-PCR. (b) Glucose uptake of AML12 cells in the blank group, NC group, and APOAV overexpression group. (c) Glut4 protein levels in the blank group, NC group, and APOAV overexpression group. ^*∗*^*P* < 0.05,  ^*∗∗*^*P* < 0.01.

As shown in Figure 6(a), compared with NC group, the *APOAV* expression was significantly upregulated (*P* < 0.01), suggesting the transfection efficiency of *APOAV* overexpression lentivirus was satisfactory. After 6 h of starvation, the glucose consumption of each group was measured by ELISA. The results showed that glucose consumption in group with *APOAV* overexpression was significantly lower than that in the NC group (*P* < 0.05; Figure 6(b)). Additionally, according to Figure 6(c), Glut4 protein level in *APOAV* overexpression group was observably increased in comparison to that in NC group with the empty vector (*P* < 0.01), which indicated that the overexpression of *APOAV* could release the insulin resistance of AML12 cells.

## 5. Discussion

In the present study, we performed the microarray profiling of insulin-resistant HepG2 cells with APOAV overexpression to investigate the differential gene expression pattern induced by APOAV overexpression in comparison to the negative controls. Previous evidence shows that insulin resistance is not only the important pathogenesis of T2D but also the root cause of various complications [25]. APOAV is a component of several lipoprotein fractions and has been reported to be negatively correlated with insulin resistance [26]. However, there have been no studies concerning the expression level of genes induced by APOAV overexpression in insulin-resistant hepatocytes. Consequently, we focused on the differential expression profiling relevant to APOAV overexpression in insulin-resistant liver cells.

### 5.1. Insulin-Resistant Cell Model Construction with HepG2 Cell

Because of the limitation of human trials, most of the fundamental research relevant to insulin resistance has applied cellular models of animals [27, 28]. HepG2 is derived from human liver embryoma cells, which show many biological properties of hepatocellular [29]. Thereby, HepG2 cell line was reliable for our research. In this paper, the insulin-resistant HepG2 cell model was firstly constructed and infected with APOAV overexpressed recombinant adenoviruses. The green fluorescence imaging illustrated that insulin-resistant HepG2 cell model was successfully infected by recombinant adenovirus, suggesting that APOAV-overexpressed HepG2 cell model was built successfully.

### 5.2. DEGs Screening and Functional Analysis

Microarray technology has been a suitable method for investigating the global gene expression in human disease, which shows an altered gene expression profiling in disease group compared with normal controls [30, 31]. Moreover, there have been studies that used microarray data for investigating gene expression levels about insulin resistance induced by mitochondrial dysfunction, reduced mitochondrial density, and increased IRS-1 serine phosphorylation [32, 33]. Besides, the DAVID gene functional classification tool can be used to condense functionally related gene list to a significant GO or pathway terms with an enrichment score. PPI network was critical to screen the significant gene nodes and understand the functions of predicted genes. Based on microarray experiments, 313 overexpressed genes and 563 low-expressed genes were identified in APOAV overexpressed cells. Hierarchical clustering analysis revealed that the B-M samples and A-Con group were clearly separated based on the differential gene expression profiles, suggesting that the differential genes identified in our paper were significant.

Furthermore, the function analysis showed that the downregulated genes were closely related with fat digestion and absorption pathway. T2D and obesity have been reported to be the metabolic diseases that are characterized by impaired insulin action and insulin resistance induced by low-grade inflammatory [34]. Recent evidence shows that nutritional fatty acids induce inflammatory response in adipocytes and macrophages [35]. Lipopolysaccharides (LPS) are another key inducer for inflammatory response during insulin resistance, which has a coreceptor for fatty acids. The plasma LPS level and LPS absorption are significantly increased during fat digestion [36]. In our study, the pathway of fat digestion and absorption were significantly related with downregulated genes in HepG2 cells induced by APOAV overexpression. Therefore, APOAV overexpression may inhibit the fat digestion and absorption triggered inflammation, and then declined the risk for insulin resistance development.

### 5.3. PPI Network and Key Genes

The PPI network is critical for understanding the functional modules for genes of interest. In this paper, two functional modules were screened out in the PPI network. Specially, genes of *AGTR1* and *P2RY2* in module 1 were found to be significantly enriched in G-protein-related signaling functions such as phospholipase C-activating G-protein-coupled receptor signaling pathway (GO:0007200), G-protein-coupled purinergic nucleotide receptor activity (GO:0045028), and G-protein-coupled receptor activity (GO:0004930). The G-protein signaling plays a key role in cell proliferation, cell wall changes, and transcriptional regulation. Besides, more than 50% drugs are developed by targeting G-protein-coupled receptors (GPCRs), revealing the critical role of G-protein signaling in various human diseases [37]. Moreover, a previous study has revealed that the insulin action is impaired by the deficiency of G-protein subunit Gi*ɑ*2 [38]. Therefore, the alteration in G-protein signaling may contribute to the progression of insulin resistance. However, the role of G-protein function has been not elucidated clearly.

*AGTR1* (degree = 11) and *P2RY2* (degree = 10) were found to be significant nodes in PPI network. *P2RY2* is one of P2Y genes and its protein production (purinergic receptor P2Y, G-protein coupled 2) is a member of GPCRs. Based on the description above, the differential expression of *P2RY2* in liver cells may contribute to the insulin resistance. *AGTR1* is the angiotensin II receptor related gene. The polymorphism of *AGTR1* is proposed to affect the insulin resistance by altering the response to angiotensin II signaling [39]. As we all know, hypertension cases are insulin-resistant; *AGTR1* polymorphism is also found to be associated with early inflammatory and metabolic changes in prehypertensive cases [40]. Besides, it is reported that the receptor of *AGTR1* is activated on liver cells in initiation of insulin resistance, which further enlarges the activation of hepatocellular NF-*κ*B signaling [41] and NF-*κ*B signaling is responsible for negative crosstalk with insulin. Although there is no evidence for the relationship between *AGTR1* and G-protein signaling, the alteration in *AGTR1* expression significantly related with insulin resistance.

It is known that glucose transporters (Gluts) play an important role in glucose regulation [42, 43]. As an insulin-regulated Glut, Glut4 mediates the glucose uptake in skeletal muscle cells. An increasing number of researches show that intracellular compartmentalization of GLUT4 is altered in models of insulin resistance [44, 45]. In this paper, cell verification experiments showed that after transfected with the *APOAV* overexpression vector, the expression of Glut4 in AML12 cell was significantly elevated, which indicated that the overexpression of *APOAV* could release the insulin resistance of AML12 cells. Moreover, *APOAV* overexpression could promote the absorption of glucose in AML12 cells. Taken together, all these evidence validated the role of *APOAV* expression in altering insulin resistance.

Furthermore, *TNFRSF1B* was a documented marker gene associated with T2D and was downregulated in liver cells induced by APOAV overexpression. TNF receptor 2 (TNFR2) encoded by *TNFRSF1B* plays a key role in insulin resistance-related metabolic disorders by mediating the metabolic effect of TNF *ɑ*. The variable genomic *TNFRSF1B* affects the soluble TNFR2 level. The level of soluble TNFR2 has been found to increase in obese patients, which is correlated with insulin resistance. In this paper, TNFRSF1B level was found to be downregulated mediated by APOAV overexpression, suggesting that APOAV overexpression inhibited TNFR2 involved inflammatory response.

In summary, APOAV overexpression may prevent the development and progression of insulin resistance in HepG2 cells by triggering differential gene expression. *APOAV* may play a key role in mediating insulin resistance by targeting *AGTR1* and *P2RY2*. Our work may provide a promising option for treating insulin-resistant metabolic diseases.

## Figures and Tables

**Table 1:** The biological function and pathways significantly related with differentially expressed genes.

	Category	Term	Count	*P* value
Up	BP	GO:0072332∼intrinsic apoptotic signaling pathway by p53 class mediator	4	3.37*E* − 03
		GO:0008283∼cell proliferation	10	1.77*E* − 02
		GO:0000278∼mitotic cell cycle	11	1.85*E* − 02
	CC	GO:0030672∼synaptic vesicle membrane	5	3.26*E* − 03
		GO:0008021∼synaptic vesicle	6	3.65*E* − 03
		GO:0031225∼anchored component of membrane	6	7.74*E* − 03
	MF	GO:0005544∼calcium-dependent phospholipid binding	5	3.06*E* − 03
		GO:0030276∼clathrin binding	4	1.65*E* − 02
		GO:0042802∼identical protein binding	13	2.68*E* − 02
	Pathway	hsa04110:cell cycle	6	6.48*E* − 03
		hsa05222:small cell lung cancer	4	4.75*E* − 02
		hsa05215:prostate cancer	4	4.89*E* − 02
Down	BP	GO:0007267∼cell-cell signaling	1.60*E* + 01	8.96*E* − 05
		GO:0007155∼cell adhesion	1.90*E* + 01	9.76*E* − 04
		GO:0070370∼cellular heat acclimation	3.00*E* + 00	2.07*E* − 03
	CC	GO:0005576∼extracellular region	65	6.66*E* − 11
		GO:0005887∼integral component of plasma membrane	51	8.76*E* − 07
		GO:0016324∼apical plasma membrane	19	6.89*E* − 06
	MF	GO:0005319∼lipid transporter activity	4	3.08*E* − 03
		GO:0008083∼growth factor activity	10	3.32*E* − 03
		GO:0046982∼protein heterodimerization activity	19	3.90*E* − 03
	Pathway	hsa05322:systemic lupus erythematosus	9	5.29*E* − 03
		hsa02010:ABC transporters	5	1.12*E* − 02
		hsa05134:legionellosis	5	2.25*E* − 02
		hsa04080:neuroactive ligand-receptor interaction	12	2.27*E* − 02
		hsa05034:alcoholism	9	2.53*E* − 02
		hsa04975:fat digestion and absorption	4	4.25*E* − 02

Up, upregulated genes; down, downregulated genes; BP, biological process; CC, cellar component; MF, molecular function.

**Table 2:** The hub nodes with highest degrees in PPI network.

Degree	Gene
19	H2AFX
18	HDAC9, ESR1
17	BMP2
15	CDC6
13	HIST2H3D, CFTR, PIK3CG
12	HIST1H4H, PDGFRB,APOE, HIST1H4I
11	AGTR1, MAPK10, HIST1H3E, HIST1H1C
10	MCM7, HMOX1, SLC2A4, POLR2A, CCKBR,SNAI2, P2RY2, H1F0

**Table 3:** List of degrees for modular genes in PPI network.

Module 1		Module 2	
Node	Degree	Node	Degree
AGTR1	11	H2AFX	19
P2RY2	10	HIST2H3D	13
CCKBR	10	HIST1H4I	12
LPAR4	9	HIST1H4H	12
F2RL2	9	HIST1H1C	11
GRPR	9	HIST1H3E	11
GPR4	8	H1F0	10
ADRA1D	8	HIST2H2BE	9
P2RY6	8	HIST2H2AA4	9
—	—	HIST1H2BD	9
—	—	HIST1H2BC	8
—	—	HJURP	1
—	—	BTG2	1

PPI: protein-protein interaction.

**Table 4:** Altered GO function and pathways for genes in significant modules.

	Category	Term	Count	*P* value
Module 1	BP	GO:0007200∼phospholipase C-activating G-protein coupled receptor signaling pathway	5	7.25*E* − 09
		GO:0007204∼positive regulation of cytosolic calcium ion concentration	4	2.29*E* − 05
		GO:0007186∼G-protein coupled receptor signaling pathway	5	4.98*E* − 04
	CC	GO:0005887∼integral component of plasma membrane	9	8.82*E* − 10
		GO:0005886∼plasma membrane	9	5.52*E* − 06
		GO:0016324∼apical plasma membrane	3	6.78*E* − 03
	MF	GO:0045028∼G-protein coupled purinergic nucleotide receptor activity	2	6.29*E* − 03
		GO:0004435∼phosphatidylinositol phospholipase C activity	2	1.35*E* − 02
		GO:0004930∼G-protein coupled receptor activity	3	3.65*E* − 02
	Pathway	hsa04080:neuroactive ligand-receptor interaction	8	1.57*E* − 10
		hsa04020:calcium signaling pathway	4	5.22*E* − 04

Module 2	BP	GO:0006334∼nucleosome assembly	11	6.08*E* − 20
		GO:0006325∼chromatin organization	6	4.58*E* − 07
		GO:0032776∼DNA methylation on cytosine	4	1.76*E* − 06
	CC	GO:0000786∼nucleosome	10	2.64*E* − 19
		GO:0000788∼nuclear nucleosome	4	2.69*E* − 06
		GO:0005654∼nucleoplasm	10	3.98*E* − 06
	MF	GO:0046982∼protein heterodimerization activity	9	1.97*E* − 10
		GO:0042393∼histone binding	6	1.26*E* − 08
		GO:0003677∼DNA binding	10	5.40*E* − 08
	Pathway	hsa05322:systemic lupus erythematosus	9	1.47*E* − 13
		Hsa05034:alcoholism	9	1.42*E* − 12
		hsa05203:viral carcinogenesis	5	8.52*E* − 05

GO, gene ontology; BP, biological process; CC, cellar component; MF, molecular function.

## Data Availability

The data used to support the findings of this study are included within the article.

## Supplementary Materials

Supplementary Materials Supplementary File 1: the detailed data information of each node in the PPI network (Figure 4).
