# Supplementary material for: The Role of Overexpressed Apolipoprotein AV in Insulin-Resistant Hepatocytes
Source: Biomed Res Int. 2020 Apr 21;2020:3268505. doi: 10.1155/2020/3268505 (PMC7193279; doi:10.1155/2020/3268505)
Supplement: Supplementary Materials — Supplementary File 1: the detailed data information of each node in the PPI network (Figure 4). [file 3268505.f1.zip › 3268505.f1/Cover Letter.docx]

Dear editor,

We would like to submit our new manuscript entitled “**The role of** **overexpressed apolipoprotein AV in insulin-resistant hepatocytes**” which we wish to be considered for publication in your journal as an Original Research article.

We announced several things as follows:

The rationale behind our study:

*APOAV seems to be closely related with insulin resistance in diabetics, but whether the overexpression of APOAV could prevent insulin resistance needs to be further determined. To explore the potential mechanism of relationship between APOAV expression and insulin resistance in hepatocytes, we firstly constructed insulin-resistant HepG2 cell model and recombinant plasmid vector with APOAV, and then infected insulin-resistant HepG2 cells by recombinant adenovirus. Sequently, transcriptome sequencing analysis was performed to identified the differently expressed genes between “the insulin-resistant HepG2 cells” and “the insulin-resistant HepG2 cells infected with the* *APOAV recombinant adenovirus vector”. Next, functional analysis and verification experiments were conducted.*

**The major findings:**

*1: APOAV overexpression may prevent the development and progression of insulin resistance in HepG2 cells by triggering differential gene expression.*

*2:* *APOAV may play a key role in mediating insulin resistance by targeting set of genes, such as AGTR1 and P2RY2.*

Thank you for any consideration of the publication of the paper!

Yours Sincerely,

Shuiping Zhao
